# Re-presentation of Olfactory Exposure Therapy Success Cues during Non-Rapid Eye Movement Sleep did not Increase Therapy Outcome but Increased Sleep Spindles

**DOI:** 10.3389/fnhum.2016.00340

**Published:** 2016-06-30

**Authors:** Julia S. Rihm, Silja B. Sollberger, Leila M. Soravia, Björn Rasch

**Affiliations:** ^1^Department of Psychology and Biopsychology, University of ZurichZurich, Switzerland; ^2^Department of Systems Neuroscience, University Medical Center Hamburg-EppendorfHamburg, Germany; ^3^Department of Psychology, Clinical Psychology and Psychotherapy, University of ZurichZurich, Switzerland; ^4^Division of Systems Neuroscience of Psychopathology, University Hospital of Psychiatry, University of BerneBerne, Switzerland; ^5^Cognitive Biology and Methods, Department of Psychology, University of FribourgFribourg, Switzerland; ^6^Zurich Center for Interdisciplinary Sleep Research, Psychiatric University Hospital ZurichZurich, Switzerland

**Keywords:** sleep, sleep spindles, targeted memory reactivation, extinction, spider phobia, arachnophobia, fear, learning

## Abstract

Exposure therapy induces extinction learning and is an effective treatment for specific phobias. Sleep after learning promotes extinction memory and benefits therapy success. As sleep-dependent memory-enhancing effects are based on memory reactivations during sleep, here we aimed at applying the beneficial effect of sleep on therapy success by cueing memories of subjective therapy success during non-rapid eye movement sleep after *in vivo* exposure-based group therapy for spider phobia. In addition, oscillatory correlates of re-presentation during sleep (i.e., sleep spindles and slow oscillations) were investigated. After exposure therapy, spider-phobic patients verbalized their subjectively experienced therapy success under presence of a contextual odor. Then, patients napped for 90 min recorded by polysomnography. Half of the sleep group received the odor during sleep while the other half was presented an odorless vehicle as control. A third group served as a wake control group without odor presentation. While exposure therapy significantly reduced spider-phobic symptoms in all subjects, these symptoms could not be further reduced by re-presenting the odor associated with therapy success, probably due to a ceiling effect of the highly effective exposure therapy. However, odor re-exposure during sleep increased left-lateralized frontal slow spindle (11.0–13.0 Hz) and right-lateralized parietal fast spindle (13.0–15.0 Hz) activity, suggesting the possibility of a successful re-presentation of therapy-related memories during sleep. Future studies need to further examine the possibility to enhance therapy success by targeted memory reactivation (TMR) during sleep.

## Introduction

Specific phobias are a form of anxiety disorder with an estimated lifetime prevalence of 11.3% in the American population (Magee et al., 1996). To date, exposure therapy is the most effective treatment for anxiety disorders (Chambless and Ollendick, 2001), especially for the fear of spiders (Öst et al., 1997b). In this approach, patients acquire a corrective experience in the phobic situation which creates a new memory trace associated with fear extinction (Bouton et al., 2006). Consolidation of this newly learned extinction memory and its integration into existing knowledge determines the reduction of symptoms which is not always given: sometimes no response to the treatment, only partial remission or relapse have been observed (Mystkowski et al., 2002, 2006).

Sleep is a promising candidate to enhance treatment outcome because of its well-known beneficial role in memory consolidation (Rasch and Born, 2013). Recognizing the high potential of sleep for the consolidation of newly acquired extinction memory in a clinical setting, studies already examined the role of sleep in fear extinction. *In sensu* exposure of spider-phobic patients by video or virtual reality treatment led to increased post-sleep fear consolidation and generalization (Pace-Schott et al., 2012) and to a higher reduction of subjective fear during approaching a spider (Kleim et al., 2014). The decrease in fear of spiders was correlated with the percentage of sleep spent in non-rapid eye movement (NREM) sleep stage 2 (Kleim et al., 2014). Additionally, NREM sleep has also been shown to play an active role in fear extinction in healthy participants (Hauner et al., 2013).

On a neural basis, spontaneous reactivations of previously learned, hippocampus-dependent information during NREM sleep are suggested as the underlying mechanism behind the memory-enhancing effect of sleep (Wilson and McNaughton, 1994). These spontaneous memory reactivations have been successfully biased in humans. For example, associating a visuo-spatial memory task with an odor and re-presenting the same odor during slow-wave sleep (SWS) leads to a better post-sleep memory performance compared with the presentation of an odorless vehicle (Rasch et al., 2007; Rihm et al., 2014). Furthermore, targeted memory reactivation (TMR) with the same odor during SWS leads to congruent odor-specific changes in electroencephalograph (EEG) activity: namely, increases in frontal delta and parietal fast spindle activity, compared with another, novel odor or an odorless vehicle (Rihm et al., 2014). However, it is still unknown whether TMR during sleep can be applied to a clinical setting to reactivate extinction memory as indicated by oscillatory changes during sleep and thereby enhance therapy success.

To test this question, spider-phobic patients underwent *in vivo* exposure-based group therapy and verbalized their subjective therapy success under the presence of a background odor. Thereafter, patients stayed awake or napped with re-presentation of the odor or presentation of an odorless vehicle. Extinction memory was tested by changes in subjective fear of spiders. We hypothesized on the behavioral level that re-presentation of the odor during sleep will lead to more pronounced extinction memory consolidation indicated by less phobia-related fear compared with an odorless vehicle or a wake interval without intervention. Additionally, we expected that odor re-presentation during sleep will result in increased delta and spindle EEG activity compared with odorless vehicle presentation.

## Materials and Methods

### Participants

Sixty medication-free spider-phobic volunteers participated in this study and were randomly assigned to the three groups: “wake without odor re-presentation”, “sleep with odor re-presentation”, and “sleep with odorless vehicle presentation”. Diagnosis of spider phobia was based on the DSM-IV (American Psychiatric Association, 2000) and assessed by the DIA-X screening questionnaires (Wittchen and Pfister, 1997; Essau et al., 1999). Exclusion criteria were general anxiety disorder, depression, mental disorders (with the exception of specific phobia), sleep-related disorders, medication intake up to 1 month prior to the experiment, major sleep disturbances or sleep rhythm changes (night shifts, shift working, time zone changes) during 8 weeks prior to the experiment, previous experience with exposure therapy, fear of moths, bad sleeping quality, and non-normal olfactory functions. In detail, exclusion criteria were determined and tested as follows: general anxiety disorder and depression were excluded via DIA-X screening questionnaires. Additionally, the likelihood of depression measured by the Beck’s depression inventory (Beck et al., 1961) did not differ between groups (*F*_(2,51)_ = 0.74, *p* > 0.40). Bad sleeping quality was assessed by the pittsburgh sleep quality index (PSQI; Buysse et al., 1989). Groups did not differ in PSQI values (*F*_(2,51)_ = 2.33, *p* > 0.14). Normal olfactory function was ensured by excluding any nasal infections at the day of the experiment, and by testing general olfactory performance using the “Sniffin’ Sticks” inventory (Burghart, Germany). One person of the odor re-presentation group had to be excluded from this general olfactory performance analysis because of missing data. The general ability to distinguish between 12 odors (mean: 11.49 ± 0.12) was comparable between groups (*F*_(2,50)_ = 0.86, *p* > 0.40). Additionally, subjective ratings on general olfactory ability from 1 (very poor) to 5 (very good) were also similar between groups (*F*_(2,51)_ = 0.03, *p* > 0.90). All other exclusion criteria were assessed by self-report.

Written informed consent was obtained after explaining the procedure. The study was approved by the ethics committee of the Faculty of Arts of the University of Zurich and in accordance with the principles of the Declaration of Helsinki (Rickham, 1964). After the first therapy session, patients were randomly assigned to three groups which differed regarding to the activity after the first therapy session. On experimental days, participants were instructed to get up at 7.00 a.m., not to take any naps and not to ingest alcohol or caffeine containing drinks.

Six participants had to be excluded from the final sample because of the following reasons: four participants showed a decrease in the behavioral approach test (BAT) of more than 3 standard deviations (SD) between two subsequent measure time points. Because of baseline differences between the three groups in the BAT, we excluded two more participants to match baselines. Furthermore, one participant was transferred from the sleep group to the wake group because her sleep time in N2 sleep was less than 5 min. Thus, our final sample consisted of 54 patients (48 female, 6 male; mean age: 25.67 ± 6.80 years (SD); range: 18–45) in the three groups: “wake without odor re-presentation group” (*N* = 18), “sleep with odor re-presentation group” (*N* = 18), and “sleep with odorless vehicle presentation group” (*N* = 18).

### Design and Procedure

**Figure 1:**
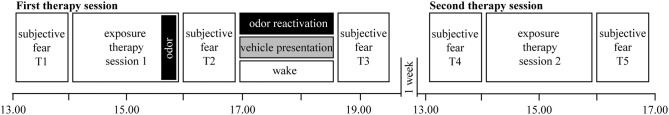
**Procedure.** Patients came to two therapy sessions separated by 1 week. At the end of the first therapy session, verbalized subjective therapy success was associated with an odor. Thereafter, patients slept or stayed awake. For the sleep groups, we presented the odor or an odorless vehicle during non-rapid eye movement (NREM) sleep. Fear was measured at five different time points: as baseline before the first exposure therapy (T1), after the first exposure therapy session (T2), after the sleep or wake interval (T3), before the second exposure therapy (T4), and after it (T5).

The study consisted of two main phases: (i) pretreatment screening via e-mail and phone; and (ii) treatment with two exposure-based group therapy sessions separated by 1 week. Before and after the two therapy sessions and after sleep or wake interventions, we assessed the fear of spiders by subjective arousal ratings and skin conductance responses (SCRs) during a picture task with spiders and moths, the distance patients could approach a living spider, questionnaires, and saliva cortisol (Figure 1).

Exposure therapy consisted of two sessions separated by 1 week and was conducted in groups of 3–4 patients by a trained psychotherapist. The exposure-based group therapy manual was adapted from the treatment manual published by Soravia et al. (2014). The first hour of both therapy sessions involved psychoeducation about spider phobia and its treatment with exposure therapy, fear circuits, avoidance behavior, and group rules. After a 5-min break, 1 h of exposure to a real spider followed. The exposures of the first session included looking at the spider in a wine glass and touching the spider in the glass with a pen. In the second therapy session, patients additionally touched the spider in the glass with a finger, caught the spider with a glass, and let the spider walk over their hand. Patients absolved each exposure under the direct guidance of the psychotherapist.

Both sessions took place between 2 p.m. and 4 p.m. and ended with a positive feedback round in which every patient reported their personal success and experienced self-efficacy during the therapy session. After the first session, subjectively experienced therapy success was associated with an odor. Then, polysomnography (PSG) was applied to patients in the sleep group, and they could nap for 90 min, whereas patients in the wake group stayed awake during this time. During stable NREM sleep stages 2 and 3, half of the sleep group was re-presented the odor of the feedback round while the other half was presented the odorless vehicle. Wake patients were free to pursue activities of their choice, but they were instructed not to take any naps or longer rests, controlled by interviews and self-reports after they returned to the lab. Analysis of the reports revealed that wake patients used the 90-min interval to go for a walk, read, do shopping, or have a tea in a nearby restaurant. Three months after the second appointment, we sent the questionnaires assessing spider phobia to all patients to measure possible time-dependent effects of the intervention interval after the first therapy session. Patients could return these questionnaires on a voluntary basis.

### Odor Delivery and Substance

The odor (magnolia and cherry blossom fragrance; Air Wick, Slough, UK) was presented during the positive feedback round at the end of therapy session 1 with the aim to associate the odor with feelings of therapy success and self-efficacy. The odor was sprayed into the room before the positive feedback round and participants smelled it during evaluating their achievements of the therapy. We chose a commercially available air freshener as odor distributor because we wanted to ensure that all the participants smelled the odor at the same time, which was not realizable with our olfactometer.

The odor was also diluted in odorless mineral oil (1:300; 1,2-propanediol; Sigma-Aldrich, Munich, Germany) in order to enable us to deliver it via an olfactometer. Since the odor was never used in previous studies we conducted pilot studies to ensure that the odor was clearly perceivable but not pungent in the used dilution. It was presented again for 30 s during sleep to the odor re-presentation group. The odorless vehicle (1,2-propanediol; Sigma-Aldrich, Munich, Germany) served as control stimulus in the vehicle presentation group, which means that it was presented the same way as the odor in the control group. Thirty seconds odor on-periods of odor presentations (in the odor presentation group), or vehicle presentations (in the vehicle presentation group), alternated with 30 s off-periods, during which room air was presented. The odor and the vehicle were delivered via a 12-channel computer-controlled olfactometer designed after Lorig (2000). Room air was filtered before entering the system, and airflow was held constant at 3 l/min. To avoid tactile or thermal shifts associated with odor onset, half of the air stream was presented continuously to the subject, and only the other half was switched between room air and vehicle or odor by computer-controlled valves. The olfactometer was placed in a separate room (adjacent to the subject’s sleeping room) and was connected to the subject’s mask via Teflon tubes, which allowed regulating the odor stimulation without disturbing the subject. The subject received the odor via a small nasal mask, which ensured constant stimulation but permitted normal breathing. We presented the odor or vehicle as soon as we detected stable N2 sleep with visible K-complexes for 2 min. Presentations continued when participants transited into N3, but were stopped immediately whenever we saw the slightest signs of arousal or rapid eye movement (REM) sleep.

### Assessment of Fear of Spiders

Fear of spiders was measured at five different time points: before (T1) and after (T2) the first therapy, after sleep or wakefulness (T3), and before (T4) and after (T5) the second therapy. Fear measures were the subjective arousal ratings and SCRs in a computer-based picture task with spider pictures, the distance to which patients could approach a living spider, subjective fear during approaching, and questionnaires about the fear of spiders. Additionally, saliva cortisol, general reaction times (RTs) and response accuracy were assessed at each time point.

#### Picture Task

In the computer-based picture task participants rated their arousal to 15 pictures of spiders and 15 pictures of moths which served as neutral control pictures (Hauner et al., 2012). Additionally, their SCRs to the pictures were recorded. To avoid habituation, five different sets of moth and spider pictures were created containing pictures that were matched with regard to size and color of the animals and background color intensity. The sets were presented in a pseudo-randomized order, whereas the 30 pictures in the set were presented randomly. After the presentation of a cross-hair for 1 s, pictures of each set were presented for 7 s with a randomized inter-stimulus interval (ISI) of 7–8 s. Participants were instructed to look at the pictures as long as possible, but if the picture became unbearable to them, they could press a key to make it disappear. After watching a picture, participants rated their arousal on a scale from 1 (very calm) to 7 (very aroused). The difference between arousal to spider and moth pictures and if participants could look at the spider pictures for the whole 7 s without skipping them were taken as fear measurements.

#### BAT and VAS

After completing the picture task, the BAT was conducted. During this task, participants had to approach a living spider in a closed box until the arousal was as high as possible but still bearable. Thus, the BAT score expresses the avoidance level towards the feared object.

Participants were accompanied to the room in which the spider was placed and had time to approach the spider slowly and at their own speed. When participants could not approach further, distance to the box that contained the spider was measured. If participants were unable to enter the room because their fear was too strong, distance to the door (600 cm) was taken as distance value.

After the BAT, the subjectively experienced anxiety during approaching the spider was rated on visual analog scales (VAS) concerning questions about the intensity of the momentary fear, the momentary feeling of physical tension, the need to leave the situation and the most catastrophic spider-related cognition by marking a cross along the 10 cm horizontal line of the scale. One participant of the odorless vehicle presentation group was excluded from the analyses of VAS data because of missing baseline data.

#### Spider Phobia Questionnaire (SPQ) and Fear of Spiders Questionnaire (FSQ)

Spider phobia at the five different time points was also assessed by German versions of the Spider Phobia Questionnaire (SPQ; Watts and Sharrock, 1984) and the Fear of Spiders Questionnaire (FSQ; Szymanski and O’Donohue, 1995). These two questionnaires were also sent to participants 3 months after the second session of the exposure therapy to assess possible time dependent changes in spider-related fear. One subject of the wake group had to be excluded from the analyses because of missing baseline data, resulting in 53 subjects for SPQ and FSQ values. Concerning the follow up, 30 out of these 53 participants sent the questionnaires back (re-presentation of odor: *N* = 11, presentation of odorless vehicle: *N* = 8, wake: *N* = 11).

#### SCRs

SCRs were recorded with Brain Vision Recorder (Munich, Germany). Data was collected from the non-dominant hand with Ag/AgCl electrodes filled with electrodermal electrode paste. Data was sampled at 250 Hz. To avoid movement artifacts, participants were instructed not to move during the whole task and the dominant hand was placed on the key with which the picture could be skipped. Before analysis, visually inspected, artifact-free data was preprocessed with MATLAB (MATLAB R2011a) by using high pass and low pass filters of 0.5 and 1.5 Hz respectively, applying a logarithmic transformation to control for the left-skewed distribution of SCR amplitudes, and by subtracting a baseline of 1 s prior to stimulus onset. Minimal and maximal points of responses were automatically detected during the presentation of the pictures, i.e., within the time frame of 1–7 s after stimulus onset. Amplitudes were calculated by subtracting the minimal from the maximal values. Finally, a range correction was performed to account for large inter-individual differences in electrodermal reactivity (Lykken et al., 1966; Boucsein et al., 2012). Pictures that were skipped by participants after their onset were treated equally to the other stimuli if no artifacts were scored by visual inspection and this SCR data was also considered between 1 and 7 s after picture onset. This was necessary because of the different picture presentation lengths depending on the time point when participants skipped the pictures. A uniform analysis would not have been possible for the mere picture presentation times, and we would have had to exclude these trials.Three participants had to be excluded from the analysis (re-presentation of odorless vehicle: *N* = 2, wake: *N* = 1) because of missing skin conductance data at T3.

#### Cortisol, General Reaction Times and Accuracy

To ensure that participants did not differ in their general state of wakefulness and vigilance, or stress, they underwent a RT and response accuracy test and gave saliva samples at all five fear measure time points T1–T5.

Cortisol samples were collected using Salivette collection tubes (Sarstedt, Germany) and stored at −20°C until analysis by the biochemical laboratory at the Institute of Psychology of the University of Zurich.

RTs were assessed by pressing a key as fast as possible whenever a big red disk appeared on a computer screen (Little et al., 1998). In 40 trials, the subjects fixed their gaze on a cross, displayed for 500–1000 ms on a white screen. In 35 trials a red disk appeared while the screen remained white in five random no-go trials. Response accuracy was calculated as the possibility to inhibit the key press to the five no-go trials.

### Sleep and EEG Recordings

Sleep was recorded by standard PSG with a 128-channel electrode cap (Geodesic, USA). Data was sampled at 500 Hz. For sleep scoring, EEG data was reduced to six scalp electrodes (F3, F4, C3, C4, P3, and P4; according to the International 10–20 System), re-referenced to contralateral mastoid electrodes, and filtered between 0.3 and 30 Hz (Iber et al., 2007). In addition to the online identification of sleep stages, EEG was scored offline by two experienced lab members according to the American Association of Sleep Medicine scoring manual (Iber et al., 2007). Sleep stages scored were wake after sleep onset (WASO), NREM sleep stages N1, N2, N3, REM sleep, and movement. For a more fine-grained exploratory analysis of effects of odor cueing during sleep, EEG recordings were subjected to spectral analyses, spindle analysis and arousal control analyses using Brain Vision Analyzer 2 (Brain Products, Germany).

#### Spectral Power Analysis

Data of the 30 s on- and off-periods of odor and vehicle stimulation were separated each into three blocks of artifact-free EEG including 10 s of data with an overlap of 10% between blocks. A Hanning window was applied on each data block before calculating power spectra using fast fourier transformation (FFT) with a resolution of 0.2 Hz. Individual mean power in the following EEG bands was determined: frontal slow delta (0.5–1.5 Hz), frontal delta (1.5–4.5 Hz), frontal slow spindle (11.0–13.0 Hz) and parietal fast spindle (13.0–15.0 Hz) bands. Data was exported as absolute power spectra. Power values were extracted from left and right frontal and parietal electrode clusters (as described in the Supplementary Material of Cordi et al., 2014). In order to take individual differences into account, the data was first normalized with an average power band between 0.5 and 50 Hz. Then, the blocks of odor on- and off-periods were used to calculate the percent change of spectral power such that power during the first 10 s interval of the odor on-period was expressed as percentage of the power during the last 10 s interval of the preceding odor off-period (set to 100%). Possible differences between hemispheres were considered by analyzing the left and the right hemisphere separately. Four participants in the sleep groups had to be excluded from the power analysis because they had fragmented NREM sleep during their nap and ended up having only one or less than one re-presentations. Nevertheless, their total sleep time and their sleep time spent in N2 sleep (>10 min) was too long to transfer them *post hoc* to the wake group. This resulted in 32 participants for the EEG analysis (*N* = 16 in the sleep with odor re-presentation group; *N* = 16 in the sleep with vehicle presentation group).

#### Spindle Analysis

For the Spindle Analysis, movement artifact-free data of the 10 s on- and off-periods after and before the odor or vehicle presentations were used. Discrete slow (11.0–12.9 Hz) and fast (13.0–14.9 Hz) spindles were identified and averaged over left and right frontal (F3 and F4) and parietal (P3 and P4) EEG recording sites (Schimicek et al., 1994; Gais et al., 2002). In brief, power was extracted in the frequency bands of interest (11.0–12.9 Hz; 13.0–14.9 Hz), and events for which the power signal exceeded a fixed amplitude threshold (±10 μV) for 0.5–3 s were counted as spindles. Movement artifact-free 10-s segments after and before odor presentation were analyzed separately for each channel (maximal difference in EMG activity of <200 μV). Data was exported and spindle counts and density were calculated in SPSS (IBM, NY, USA). We calculated spindle counts as the sum of the number of spindles in the 10-s on-periods after odor or vehicle onset relativized with the sum of the number of spindles in the 10-s off intervals. Thus, we obtained a change in percent in the number of spindles before compared with after the odor or vehicle presentation onset. To calculate spindle density, spindle counts for the on- and off-periods were divided by the number of analyzed 10-s epochs and the values for the on-periods were relativized with the values for the off-periods, also resulting a change in percent from before compared with during the odor or vehicle presentation. Two participants (one in the sleep with odor re-presentation, one in the odorless vehicle presentation group) had to be excluded from this spindle analysis because EMG electrodes fell off during the nap and the EMG criterion was not applicable. We had to exclude three more participants (two in the sleep with odor re-presentation, one in the odorless vehicle presentation group) for the spindle analysis over parietal electrodes because they had no spindles at all in the analyzed off-segments. Therefore, a calculation of the count and density values by division of the on- by the off-values was not possible.

#### EEG Arousal Control

To control for possible arousals in response to odor presentations, we visually scored the arousals in the EEG data during 10 s after the odor onset. We used the arousal scoring rules of the American Sleep Disorders Association (Bonnet et al., 1992). According to these rules, arousals were scored when EEG data shifted for at least 3 s to a higher frequency (theta, alpha, and/or >16 Hz) in NREM sleep.

### Statistical Analysis

EEG data was analyzed by repeated measures analysis of variances (ANOVAs) with repetition in the factor “lateralization” (left vs. right hemisphere) between sleep groups (odor re-presentation vs. odorless vehicle presentation). Significant interactions were further analyzed by *post hoc* *t*-tests between the two groups.

For behavioral data analyses, we proceeded similar as Kleim et al. (2014). We focused on the differences between groups between T2 and T3 (immediately after the first therapy and after the sleep or wake interval) and between T3 and T4 (after the sleep or wake interval at the end of the first session and before the second session 1 week later) separately by calculating absolute and relative values. Therefore, we subtracted data at T2 from T3, and at T3 from T4. For relative changes, data of T3 was divided by T1 and multiplied by 100. Similarly for the second session, for relative values data of T4 was divided by T3 and multiplied by 100. Calculation of relative changes was not possible for the BAT distance scores as most patients reached a distance of 0 cm at T3. These absolute and relative values were compared by one-way ANOVAs between the three groups.

In order to show the general impact of extinction learning on fear of spiders, we focused only on the therapy sessions and analyzed data by using a 2 × 2 × 3 ANOVA with the repeated measures factors session (first vs. second session) and time (pre vs. post therapy) between groups (odor re-presentation during sleep vs. vehicle presentation during sleep vs. wake). Additionally, chi square tests were conducted to analyze if groups differed in skipping spider pictures at T3 and T4. A *p* value < 0.05 two-tailed was considered significant.

## Results

### Fear of Spiders

In general, exposure therapy was highly efficient and significantly reduced the subjects’ fear of spiders as measured by approach behavior (BAT), subjective fear ratings (VAS), spider phobia questionnaires (SPQ, FSQ), electrodermal activity (SCRs), and subjective arousal during exposure to spider pictures. In particular, we found a main effect of time (before and after the therapy; all *p* < 0.001) and session (first and second therapy; all *p* < 0.001) as well as significant time by session interactions (all *p* ≤ 0.02; except for FSQ: *p* = 0.08) for all of these measures. These effects indicated a decrease in fear after the therapy sessions compared with before the sessions, and a decrease in fear from the first to the second session. The interaction reflected a stronger reduction of fear during the first than during the second session.

Despite the great efficacy of our therapy concerning symptom reduction in general in all three groups, we observed no evidence that odor re-presentation during sleep or sleep in general improved therapy success compared with the wake control. The results of these parameters are listed below. A Bonferroni-corrected bivariate correlation analysis between each relative and absolute behavioral parameter and the number of reactivations in the odor re-presentation group did not reveal any significant results (all *r* between −0.55 and 0.40, all *p* > 0.05).

#### BAT and VAS

**Figure 2:**
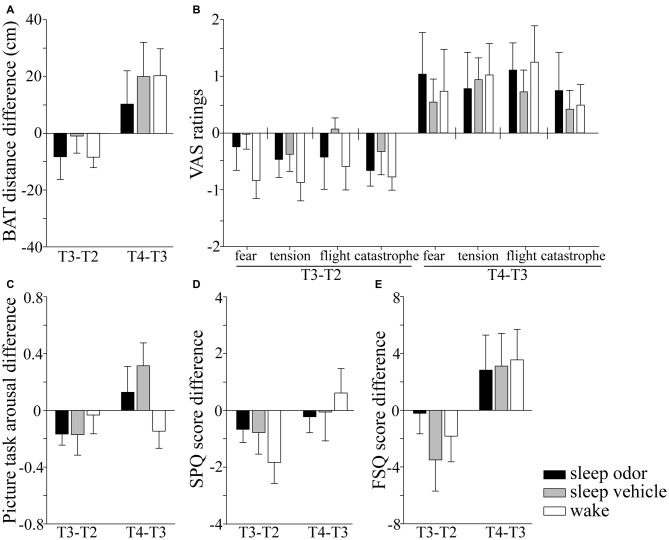
**Absolute changes in fear parameters from time point T2 (after the first exposure therapy) to T3 (after sleep/wake) and from T3 (after sleep/wake) to T4 (1 week later), for the following measures: (A) behavioral approach test (BAT), (B) fear during BAT measured by four different visual analog scales (VAS) for momentary fear (fear), momentary feeling of physical tension (tension), need to leave the situation (flight) and catastrophic spider-related cognitions (catastrophe), (C) the differences in subjective arousal between moth and spider pictures, (D) scores for the spider phobia questionnaire (SPQ), and (E) scores for the fear of spiders questionnaire (FSQ).** Negative values indicate a decrease in fear compared with the time point before. Data are Mean ± SEM.

The ability to approach a living spider was not influenced by sleep or odor re-presentation during sleep compared with wake (*F*_(2,51)_ = 0.48, *p* > 0.60), and differences between the intervention groups were also not visible 1 week later (*F*_(2,51)_ = 0.26, *p* > 0.70; Figure 2A). There were also no differences between groups when analyzing the differential changes between each of the succeeding pairs of measure time points (all *p* > 0.50; Table 1). Absolute and relative VAS ratings in all four scales did also not differ between the three groups at T3 (all *p* > 0.20) or at T4 1 week later (all *p* > 0.40; Figure 2B).

**Table 1:** **Baseline values and differential changes in distance in cm during the behavioral approach test (BAT) between each pair of succeeding time points T1 (beginning of the first session), T2 (after the first exposure therapy), T3 (after the sleep/wake interval), T4 (1 week later, beginning of second session) and T5 (after the second exposure therapy) and for the decrease between the two therapy sessions**.

Time point	Odor (*N* = 18)	Odorless vehicle (*N* = 18)	Wake (*N* = 18)	*F*_(2,51)_	*p*point
T1	123.56 ± 26.80	119.17 ± 32.45	93.17 ± 18.05	0.39	0.68
T2-T1	−90.22 ± 24.13	−87.01 ± 23.74	−59.78 ± 14.84	0.62	0.54
T3-T2	−8.28 ± 8.04	−1.00 ± 6.03	−8.50 ± 3.66	0.48	0.62
T4-T3	10.28 ± 11.71	20.00 ± 12.02	20.28 ± 9.51	0.26	0.77
T5-T4	−29.83 ± 7.86	−30.94 ± 15.69	−33.44 ± 42.14	0.03	0.98
T5-T2	−27.83 ± 6.92	−11.94 ± 11.55	−21.67 ± 27.12	0.87	0.43

*Negative values indicate a closer approach to the spider compared with the time point before. Data are Means ± SEM. Right columns indicate F and p values for one-way ANOVAs*.

#### Picture Task

Similarly, the difference in subjective arousal towards phobic and non-phobic pictures was the same for the three groups at T3 (*F*_(2,51)_ = 0.42, *p* > 0.60), and 1 week later at T4 (*F*_(2,51)_ = 2.21, *p* = 0.12). Groups did also not differ in the duration of looking at the spider pictures at T3 (*χ*^2^_(2,52)_ = 1.04, *p* > 0.50) or T4 (*χ*^2^_(2,52)_ = 1.66, *p* > 0.40; Figure 2C).

#### SPQ and FSQ

None of the questionnaires revealed differences between the three groups at T3 (all *p* > 0.15) or T4 (all *p* > 0.70) in absolute or relative values (Figures 2D,E). Three months after the therapy, the three groups did still not differ in their absolute or relative values in FSQ and SPQ scores compared with the baseline before the first (all *p* > 0.17) or after the second therapy session (all *p* > 0.30).

#### SCRs

At every time point T1–T4, patients revealed higher physiological reactivity to spider compared with moth pictures, independent of group (all *p* < 0.005). However, after the second therapy at T5, the SCRs to spiders and moths were comparable (*F*_(2,48)_ = 1.49, *p* > 0.20). The SCRs of the three groups were comparable at all time points (all *p* > 0.12).

#### Cortisol, General RT and Accuracy

The three groups did not differ in absolute nor in relative changes in cortisol values at T3 (both *p* > 0.20) or T4 (both *p* > 0.40). The three groups did also not differ in general RT or response accuracy after sleep or wakefulness at T3 (both *p* > 0.70), nor at T4 (both *p* > 0.40).

### Sleep and Re-Presentation of Therapy Success

#### Sleep Architecture

Neither total sleep time nor percentage of WASO, N1, N2, N3, and movement nor number of olfactory stimuli presentations differed between the two sleep groups (all *p* > 0.14; Table 2). All odor re-presentations during sleep were placed in sleep stages N2 or N3 (100% correct placement for every patient in both groups). The number of re-presentations ranged from 12–65 in the odor presentation and from 6–59 in the vehicle presentation group. Only 10 out of 36 patients reached REM sleep (odor re-presentation: *N* = 4; vehicle presentation: *N* = 6). Their percentage in REM sleep did also not differ (*χ*^2^_(1,9)_ = 10.00, *p* > 0.30).

**Table 2:** **Wake after sleep onset (WASO), non-rapid eye movement (NREM) sleep stages 1 (N1) and 2 (N2), slow-wave sleep (SWS) (N3), and rapid eye movement (REM) sleep in % of total sleep time (total time)**.

Sleep parameters	Odor (*N* = 18)	Odorless vehicle (*N* = 18)	*t*_(34)_	*p*
WASO (%)	11.41 ± 2.55	18.92 ± 4.37	1.49	0.15
N1 (%)	15.26 ± 1.76	12.59 ± 1.72	−1.08	0.29
N2 (%)	47.26 ± 4.70	41.52 ± 3.60	−0.97	0.34
N3 (%)	24.50 ± 5.66	23.22 ± 4.34	−0.18	0.86
REM (%) ^a^	4.27 ± 0.66	8.97 ± 2.18		0.35
Movement (%)	0.63 ± 0.23	0.72 ± 0.25	0.24	0.81
Total time (min)	92.61 ± 7.03	84.50 ± 7.24	−0.80	0.43
Number stimulations^b^	28.56 ± 3.69	35.56 ± 4.36	−1.23	0.23

*^a^Only 10 out of the 36 patients reached REM sleep (reactivation with odor: N = 4; presentation of odorless vehicle: N = 6; df = 9; χ^2^ test). ^b^Two of the sleep participants in each sleep group did not reach stable N2 sleep and therefore only one or less than one reactivations. These participants are excluded from the EEG analysis and from the number of reactivation statistics, resulting in 16 participants per group. Right columns indicate t and p values for t tests. Data are Means ± SEM*.

#### Effects of Cued Odor Presentations on Oscillatory Activity

In line with our hypothesis, EEG activity in the spindle bands during the first 10 s of odor re-presentation in NREM sleep induced by the same odor presented at the end of extinction learning differed from EEG activity during presenting an odorless vehicle.

**Figure 3:**
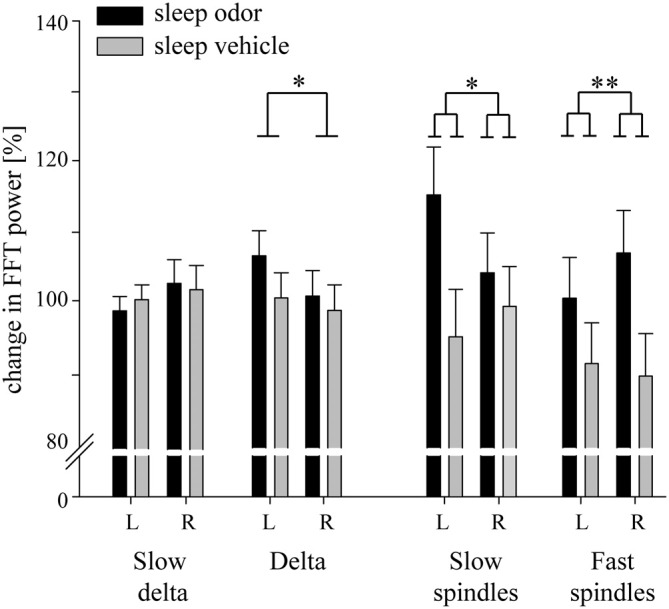
**Changes in relative electroencephalograph (EEG) activity during the first 10 s of odor on-intervals compared with the last 10 s of odor off-intervals.** Data for slow delta (0.5–1.5 Hz), delta (1.5–4.5 Hz) and slow spindle (11.0–13.0 Hz) activity are retrieved from frontal electrodes. Data for fast spindle activity (13.0–15.0 Hz) are retrieved from parietal electrodes. L: left hemisphere, R: right hemisphere. Data are Means ± *SEM*. *p* values from one-way ANOVAs are indicated (*p < 0.05, **p < 0.01).

**Figure 4:**
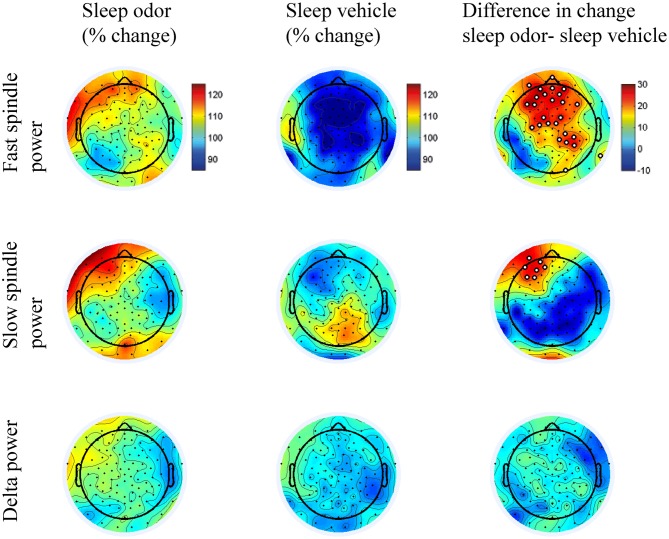
**Scalp distributions of the sleep odor and sleep vehicle groups.** Figures illustrate the scalp distributions in the fast (13.00–15 Hz) and slow (11.0–13.0 Hz) spindle bands and in the delta band (1.5–4.5 Hz). Data is indicated for the percentage change from the 10-s off interval to the 10-s on interval for the sleep with odor re-presentation and the sleep with vehicle presentation groups. The last column illustrates the difference in scalp distributions between the two groups. We conducted a *t* test for each electrode site between the two groups and marked electrodes in white in the difference plots if the *p* value was < 0.05.

Over the right parietal hemisphere, the interaction between group and laterality was highly significant (*F*_(1,30)_ = 10.93, *p* = 0.002) for relative changes in fast spindle activity (13.0–15.0 Hz) from odor off- to odor on-periods. Moreover, these changes were higher in the odor re-presentation group (106.86 ± 6.05%) compared with the vehicle group (89.27 ± 6.05%, *t*_(30)_ = 2.06, *p* = 0.049). No difference occurred in the left parietal hemisphere (odor re-presentation: 100.35 ± 5.84% vs. vehicle presentation: 91.02 ± 5.84%; *t*_(30)_ = 1.13, *p* > 0.2; Figures 3, 4). The differences in relative fast spindle power changes were also significantly pronounced over non-lateralized frontal sites, as revealed by a scalp distribution (Figure 4). There was no overall difference between the two sleep groups (*p* = 0.11).

Interestingly, a lateralization was also observed in the slow spindle band (11.0–13 Hz). Again, the interaction between odor re-presentation group and laterality was significant (*F*_(1,30)_ = 6.65, *p* = 0.02). Patients with odor re-presentation during sleep revealed higher changes in slow spindle activity over the left frontal hemisphere (115.18 ± 6.80%) compared with the odorless vehicle group (94.87 ± 6.80%, *t*_(30)_ = 2.11, *p* = 0.04). No difference occurred over the right frontal hemisphere (odor re-presentation: 104.05 ± 5.71% vs. vehicle presentation: 99.25 ± 5.71%, *t*_(30)_ = 0.59, *p* > 0.50; Figures 3, 4).

The finding of increased spindle activity after odor re-presentation was paralleled by distinct sleep spindles: changes in fast parietal spindle counts were distinctly enhanced in the odor re-presentation compared with the odorless vehicle group, although no effect of lateralization was observed (odor re-presentation: 172.84 ± 24.54% vs. vehicle presentation: 95.33 ± 25.47%; *F*_(1,25)_ = 4.80, *p* = 0.04). A similar pattern was observed for changes in frontal slow spindle counts (135.93 ± 8.44% vs. 98.67 ± 8.44%; *F*_(1,28)_ = 9.74, *p* = 0.004). Changes in spindle density were also higher after odor presentation compared with odorless vehicle presentation for frontal slow spindles (odor presentation: 108.74 ± 5.27%, odorless vehicle presentation: 91.66 ± 5.27%; *F*_(1,28)_ = 5.25, *p* = 0.03) and showed a statistical trend for parietal fast spindles (odor re-presentation: 142.40 ± 21.18%, odorless vehicle presentation: 88.31 ± 21.98%; *F*_(1,25)_ = 3.14, *p* = 0.09). In these analyses, neither a lateralization main effect nor a lateralization by condition interaction was found.

Contrary to the two spindle bands, we observed no effect of odor re-presentation in frontal delta activity, neither as interaction with laterality nor as main effect of group (both *p* > 0.20; Figure 3). Independent of group, frontal delta activity (1.5–4.5 Hz) over the left hemisphere (103.46 ± 2.53) was significantly higher than over the right hemisphere (99.67 ± 2.60; *F*_(1,30)_ = 4.95, *p* = 0.03). Descriptively, the decrease was higher in the odor re-presentation group (Figure 3). The re-presentation had no impact on frontal slow delta power (0.5–1.5 Hz, all *p* > 0.06). Importantly, odor presentation did not result in changes in EEG arousals after odor onset compared with odorless vehicle presentation (*t*_(30)_ = 0, *p* = 1.0; odor re-presentation: mean = 1.38 ± 0.30; vehicle presentation: mean = 1.38 ± 0.30).

## Discussion

Our results show that re-presentation of an odor previously cued with subjectively experienced therapy success during NREM sleep did not result in differences in therapy outcome between patients that were re-presented the odor, patients that were re-presented an odorless vehicle, and patients that did not sleep at all. However, we found that re-presenting the previously cued odor during NREM sleep leads to increased frontal slow and parietal fast spindle activity compared with the presentation of an odorless vehicle. But, contrary to our hypothesis, these changes in EEG activity were not present in the delta band. Thus, our findings indicate that re-presentation of an odor which was previously associated with subjective therapy success is visible in changes in electrophysiological spindle activity, but that *in vivo* exposure group therapy might cause a ceiling effect in extinction learning and thus extinction memory could not be further improved by sleep or odor re-presentations during sleep. Alternatively, cueing of the verbalized therapy success could have been not potent enough to induce therapy success.

In our previous work we could show that EEG activity in response to TMR with a different odor than during learning was comparable with odorless vehicle presentation (Rihm et al., 2014). Therefore, we can assume that effects on spindles in this study are due to the presentation of the memory-associated cue rather than mere effects of sleep preservation in response to environmental stimuli and thus we included only an odorless vehicle presentation group in this study. However, please note that we could show this effect only in healthy participants, and we can only speculate that the spindle responses to non-cued odors are similar in anxiety patients. Furthermore, previous studies support the notion of a critical role of NREM sleep in fear extinction (Hauner et al., 2013; Kleim et al., 2014). The finding that TMR with the same odor presented during learning alters EEG activity during NREM sleep is in line with this notion and with our previous findings (Rihm et al., 2014), although in the present study, this effect depended on lateralization. Our results suggest that the neural mechanisms of fear extinction consolidation during NREM sleep might be similar to those of declarative memory consolidation, where thalamo-cortical spindles during SWS sleep establish a dialog between the hippocampus and neocortex, enabling information transfer between these brain regions (Mölle and Born, 2011). The asymmetry in terms of left lateralization for slow spindle activity and right lateralization for parietal fast spindle activity during olfactory TMR is a new finding. A possible explanation for the lateralization is the strong emotional involvement while verbalizing subjective therapy success. On the one hand, increases of left-lateralized frontal EEG activity have been associated with tasks that induce a good mood (Davidson and Fox, 1982; Ekman and Davidson, 1993) and with greater trait approach motivation (Coan and Allen, 2003; Amodio et al., 2008). In our study, good mood, and a motivation to approach the feared object exactly describe the patients’ state after verbalizing subjective therapy success and associated self-efficacy. On the other hand, right posterior regions have been involved in the processing of emotional stimuli. For example, both positive and negative ERP components to emotional stimuli are larger over right parietal regions (Kayser et al., 1997; Keil et al., 2001; Thomas et al., 2007). Importantly, we can exclude arousals or awakenings in the reactivation group as a possible explanation for the observed changes in spindle spectral power.

The results of our study do not support the findings of previous studies that sleep benefits the enhancement of extinction memory on a behavioral level and thus reduces fear after exposure. In contrast to our *in vivo* exposure-based group therapy, these studies used *in sensu* therapy with a virtual environment (Kleim et al., 2014) or a short movie clip (Pace-Schott et al., 2012). A reason for the missing behavioral effects of sleep on extinction memory consolidation after our *in vivo* exposure therapy could be a possible ceiling effect of extinction learning. Exposure therapy has been shown to be a very effective treatment, even after one session (Öst, 1996; Öst et al., 1997a,b) and in particular for spider phobia (Choy et al., 2007; Soravia et al., 2014). Since we applied a well-established experimental effect to a real psychotherapeutical situation in a group format, it was not possible to create a highly controlled experimental setting as for example in studies investigating the effect of sleep on declarative memory, where it is common to define a fixed learning criterion, e.g., 60% of the overall learning material (Rasch et al., 2007; Rihm et al., 2014) or the placement within a certain diameter from the target stimulus (Rudoy et al., 2009).

Due to the absence of immediate effects of sleep on treatment outcome after a virtual reality treatment (Kleim et al., 2014), we measured fear again 1 week after the initial therapy. However, the different sleep groups and the wake group did also not differ at this time point, nor did they 3 months later. Importantly, we can rule out different vigilance states between the groups, especially after the sleep intervention, on behavioral results by comparing RT tests and correct responses in a go–no go test at every time point.

Since we applied a group therapy setting, it was not possible to individually associate the extinction learning process with the odor. Anxiety in the observing patients—beside the acting patient—would eventually have led to the association of fear with the odor. Moreover, after successful odor-extinction association, other patient’s subsequent extinction trials with presentation of the odor could have weakened the new memory trace, since TMR on declarative memory during wakefulness led to a destabilization of the newly acquired memory trace (Diekelmann et al., 2011). However, we cannot rule out the possibility that cueing only the verbalized therapy success—which is rather abstract compared with specific actions during extinction learning—could have been too weak to enhance extinction memory. Taken together, re-exposure to *in vivo* exposure-based group therapy for spider phobia during NREM sleep leads to lateralized increases in slow and fast spindle bands. This impact on the EEG could be reflected by reduced fear of spiders, if exposure learning is not already at ceiling. For future studies we suggest using other extinction learning paradigms, e.g., *in sensu*, to further explore the present finding.

## Author Contributions

JSR, LMS, and BR: designed the experiment, JSR and SBS: acquired data, JSR, LMS, and BR: analyzed the data, JSR, SBS, LMS, and BR: interpreted the data, JSR, SBS, LMS, and BR: drafted and revised the work critically for important intellectual content, JSR, SBS, LMS, and BR: finally approved the version to be published, JSR, SBS, LMS, and BR: agreed to be accountable for all aspects of the work in ensuring that questions related to the accuracy or integrity of any part of the work are appropriately investigated and resolved.

## Conflict of Interest Statement

The authors declare that the research was conducted in the absence of any commercial or financial relationships that could be construed as a potential conflict of interest.
